# Supplementary material for: Microbial production of megadalton titin yields fibers with advantageous mechanical properties
Source: Nat Commun. 2021 Aug 30;12:5182. doi: 10.1038/s41467-021-25360-6 (PMC8405620; doi:10.1038/s41467-021-25360-6)
Supplement: Supplementary file 1 — Supplementary Information [file 41467_2021_25360_MOESM1_ESM.pdf]

Supplementary Information for
Microbial production of megadalton titin yields fibers with advantageous mechanical properties

Christopher H. Bowen[1,#], Cameron J. Sargent[2,#], Ao Wang[3], Yaguang Zhu[1], Xinyuan Chang[1], Jingyao Li[1], Xinyue Mu[1], Jonathan M. Galazka[4], Young-Shin Jun[1], Sinan Keten[3], Fuzhong Zhang[*,1-2,5]

[*]Correspondence to: fzhang@seas.wustl.edu

This PDF file includes:

Supplementary Figures 1-13

Supplementary Tables 1-8

Supplementary Notes 1-4

Supplementary References (1-33)

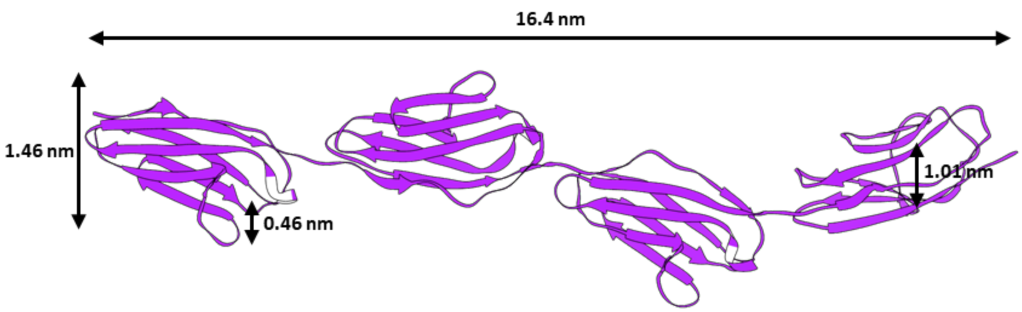

Supplementary Fig. 1. Dimensions from the titin 4Ig crystal structure.[1] Dimensions indicated are the Ig domain width (1.46 nm), inter-sheet distance (1.01 nm), inter-chain distance (0.46 nm), and 4Ig length (16.4 nm). Approximate dimensions were determined by analysis of the 3B43 structure from the Protein Data Bank using UCSF Chimera.[1] Source data are provided as a Source Data file.

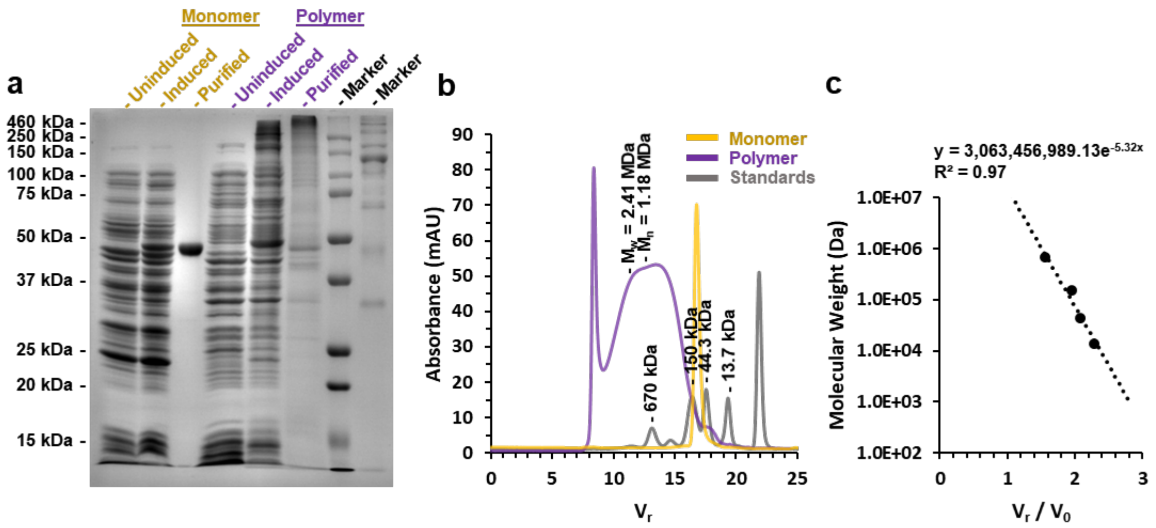

Supplementary Fig. 2. Production and purification of titin monomer and polymer. (a) 12% SDS-PAGE gel of uninduced and induced total cell lysates and HisTrap purified protein. Similar gel images were acquired throughout protein production, purification, and analysis. **(b)** Analytical SEC A280 chromatograms for monomer (gold), polymer (purple), and MW standards (gray; see Methods). **(c)** SEC calibration curve acquired as described in methods. Source data are provided as a Source Data file.

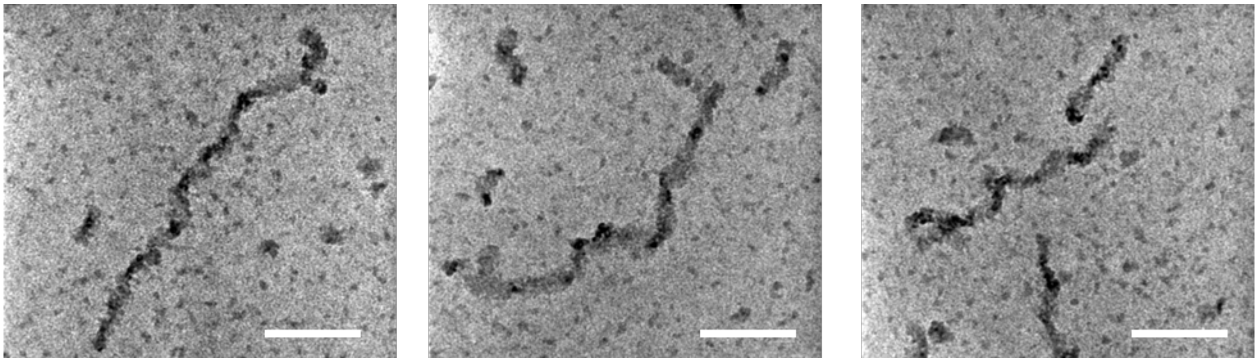

Supplementary Fig. 3. STEM analysis of purified UHMW titin polymer. Dark-field negative stain STEM images of purified titin polymers, representative of dozens of similar polymers observed using STEM and among the 42 polymers that were selected for diameter measurements. Scale bars are 50 nm. Eluents from the HisTrap column were fully dialyzed against 5 mM ammonium bicarbonate and prepared for imaging as described in methods.

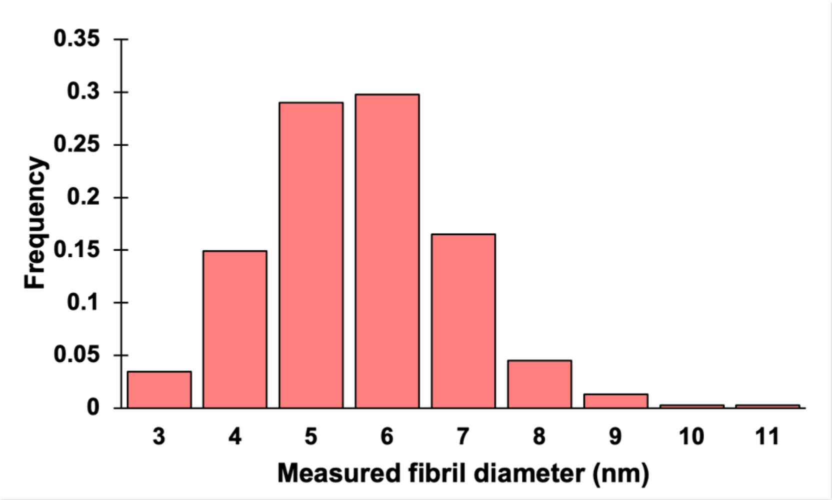

Supplementary Fig. 4. Distribution of UHMW titin polymer diameters measured by STEM. Fibrils had an average cross-sectional diameter of 6.1 nm (± 1.2 nm, n = 376, where n is total number of measurements across multiple fibrils), which is consistent with fibril diameters found in previous studies (8-10 nm, see SI Figure 5 in the referenced paper).[1] Source data are provided as a Source Data file.

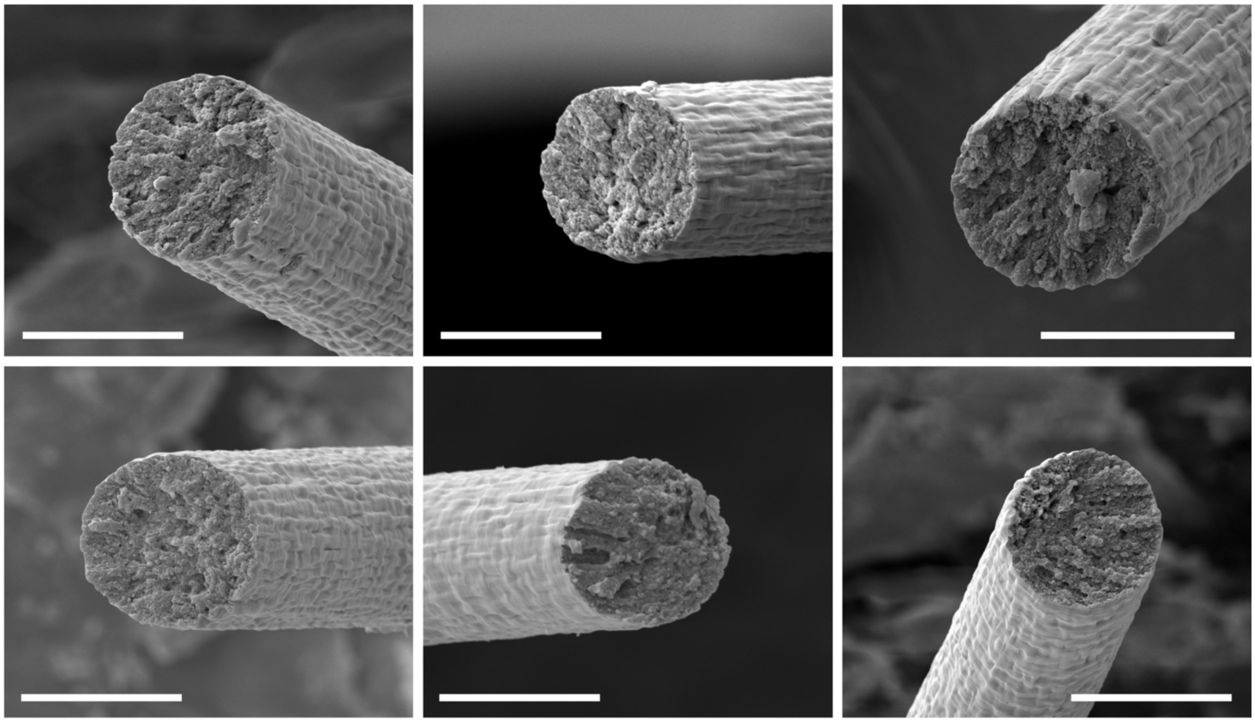

Supplementary Fig. 5. SEM images of microbially produced UHMW titin fibers. Fibers were imaged after pull tests as described in methods. Scale bars are 10 µm. Fibers showed highly consistent size and morphology between samples. These images are representative of 12 fibers that were imaged using SEM.

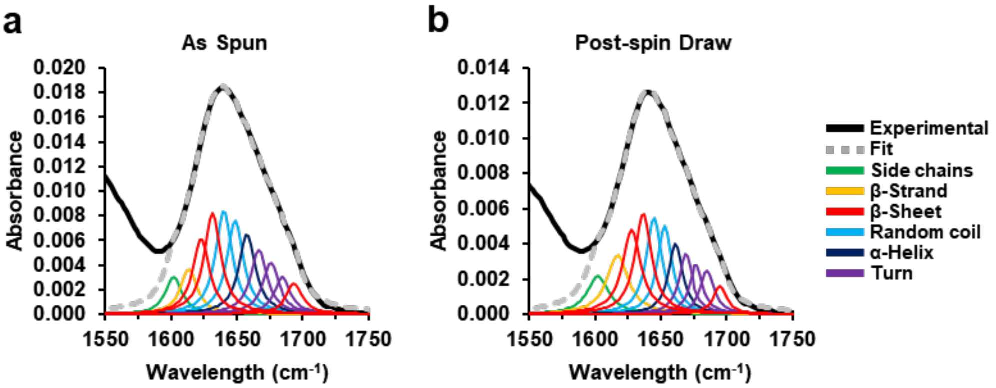

Supplementary Fig. 6. FTIR analysis of microbially produced UHMW titin fibers. Using representative FTIR spectra from as-spun **(a)** and post-spin drawn **(b)** fibers, amide I bands were deconvolved into sets of 11 Lorentzian peaks based on previously reported methods (see Methods). Source data are provided as a Source Data file.

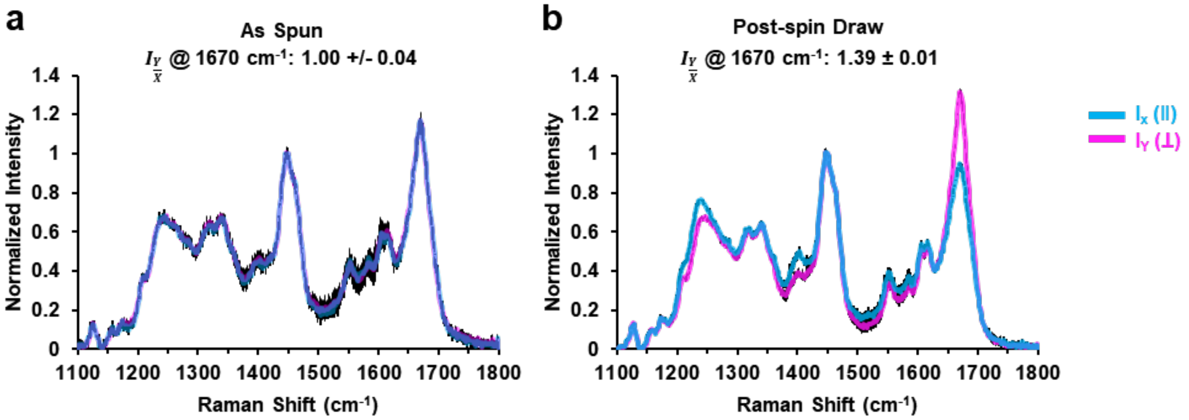

Supplementary Fig. 7. Raman spectromicroscopy analysis of as-spun (a) and post-spin drawn (b) microbially produced UHMW titin fibers. Raman spectra were acquired for fibers oriented perpendicular (pink line) or parallel (blue line) to the polarization of the incident laser. Spectra were normalized to the intensity of the peak at 1460 cm^{-1}, which corresponds to orientation insensitive CH_2 bending (see Methods). Spectra shown are the average of spectra acquired on three separate fibers for each fiber state. Standard deviations of the three measurements at each Raman shift are shown as black bars. As a measure of orientation sensitivity, the average ratio of the amide I peak (1670 cm^{-1}) intensity at 0° to that at 90° is shown above each spectrum. Black vertical lines represent the standard deviation of the three measures at each Raman shift. Source data are provided as a Source Data file.

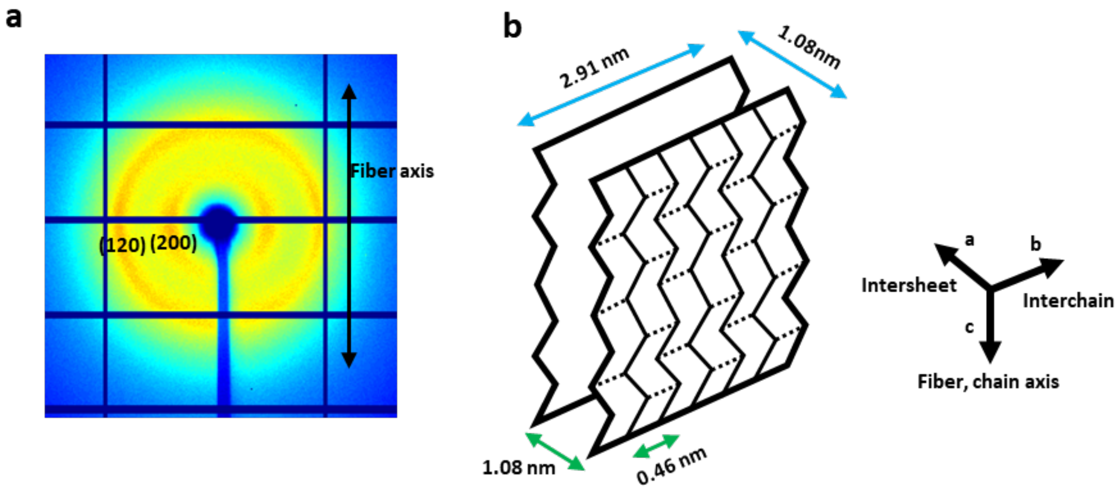

Supplementary Fig. 8. Microbially produced UHMW titin fiber WAXD data and interpretation. (a) 2D WAXD image for UHMW titin fiber, revealing two broad, but distinct equatorial reflections perpendicular to the fiber axis, along with a substantial amorphous component characteristic of a semi-crystalline material. Assuming an orthorhombic unit cell commonly applied to β-sheet crystallites in semi-crystalline fibers, the innermost equatorial peak is indexed as (200), corresponding to inter-sheet d-spacing along the unit cell a-axis, and the outermost equatorial peak is indexed as (120), corresponding to inter-chain d-spacing along the unit cell b-axis. **(b)** The resulting center positions of the (200) and (120) crystalline peaks indicate a-axis inter-sheet d-spacing of 1.08 nm and b-axis inter-chain d-spacing of 0.46 nm, respectively. From the center position and FWHM of the (200) and (120) peaks, the Scherrer equation was used to determine the average crystallite size of 1.08 nm along the inter-sheet a-axis and 2.91 nm along the inter-chain b-axis, respectively (see Methods; green lines indicate d-spacings, blue lines indicate calculated average crystallite sizes).

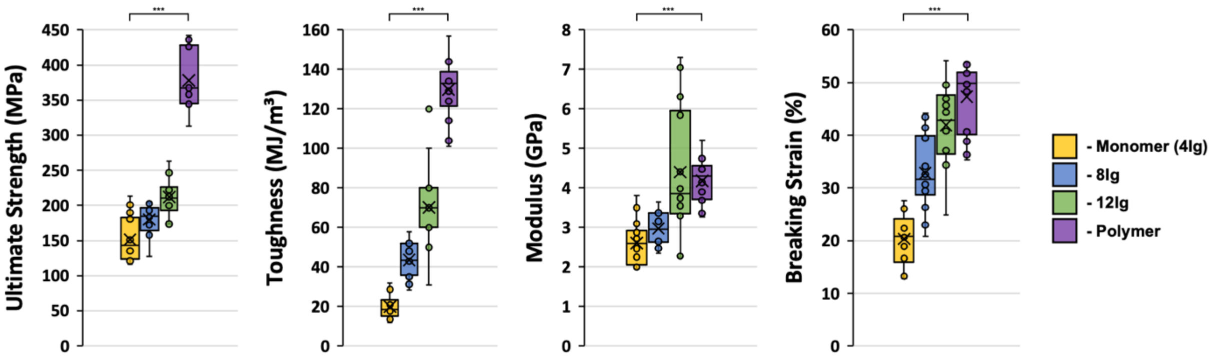

Supplementary Fig. 9. Mechanical properties of fibers spun from different sizes of titin. Ultimate tensile strength, toughness, elastic modulus, and breaking strain as calculated from tensile tests of fibers made from 4Ig monomer (gold), 8Ig (blue), 12Ig (green), and UHMW polymer (purple) proteins (n=14; horizontal lines denote, from top to bottom, upper fence, Q3, median, Q1, and lower fence; × denotes mean; other data indicated with circles). *** Unpaired two-tailed t-test $P = 4.4 \times 10^{-15}$, 1.6×10^{-19}, 1.1×10^{-7}, and 2.1×10^{-12} for the ultimate tensile strength, toughness, modulus, and breaking strain, respectively. Source data are provided as a Source Data file.

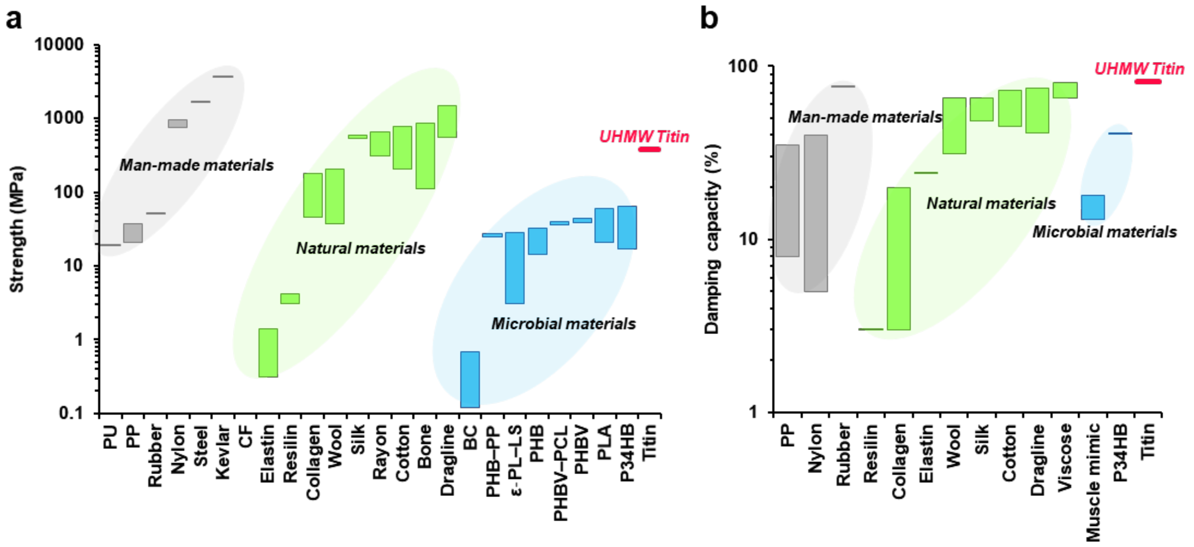

Supplementary Fig. 10. Strength (a) and damping capacity (b) of the microbially produced titin fiber compared to other man-made (grey), natural (green), and microbially produced (blue) materials (see Supplementary Table 8). Source data are provided as a Source Data file.

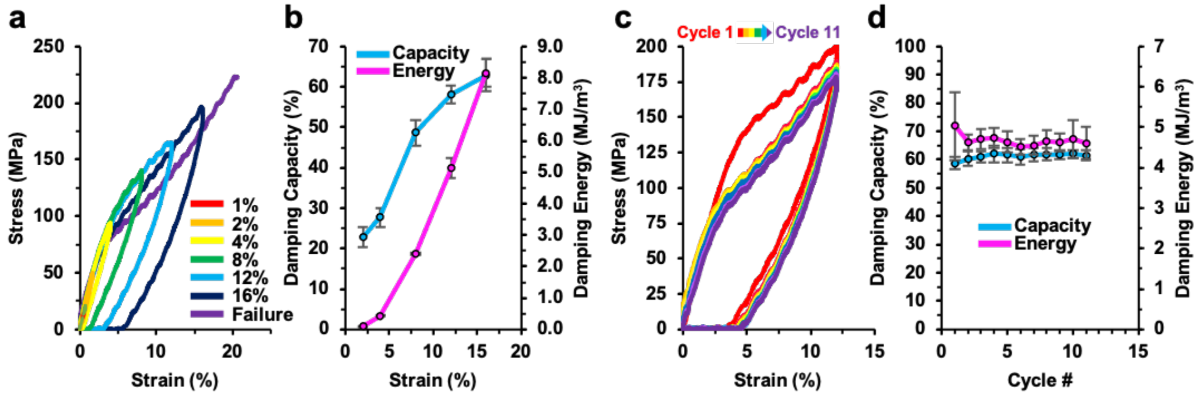

Supplementary Fig. 11. Damping capacity and mechanical recovery of fibers spun from titin monomer. (a) Loading/unloading curves for 4Ig titin (monomer) fibers acquired at increasing strains from 1-16%. **(b)** Average calculated damping capacity (blue curve) and damping energy (pink curve) at each strain tested in (a). Error bars are the standard deviation of the three fiber samples tested at each strain. **(c)** Stress-strain curves for 4Ig titin (monomer) fibers subjected to 11 consecutive loading/unloading cycles with one minute of humid (95% RH) air treatment between cycles. Stress-strain curve of the first round is colored red. Following cycles use other colors. **(d)** Average calculated damping capacity (blue curve) and damping energy (pink curve) of monomer titin fibers over consecutive cycles shown in (c). Error bars are the standard deviation of the values measured at each cycle number for the three fiber samples that were tested. Source data are provided as a Source Data file.

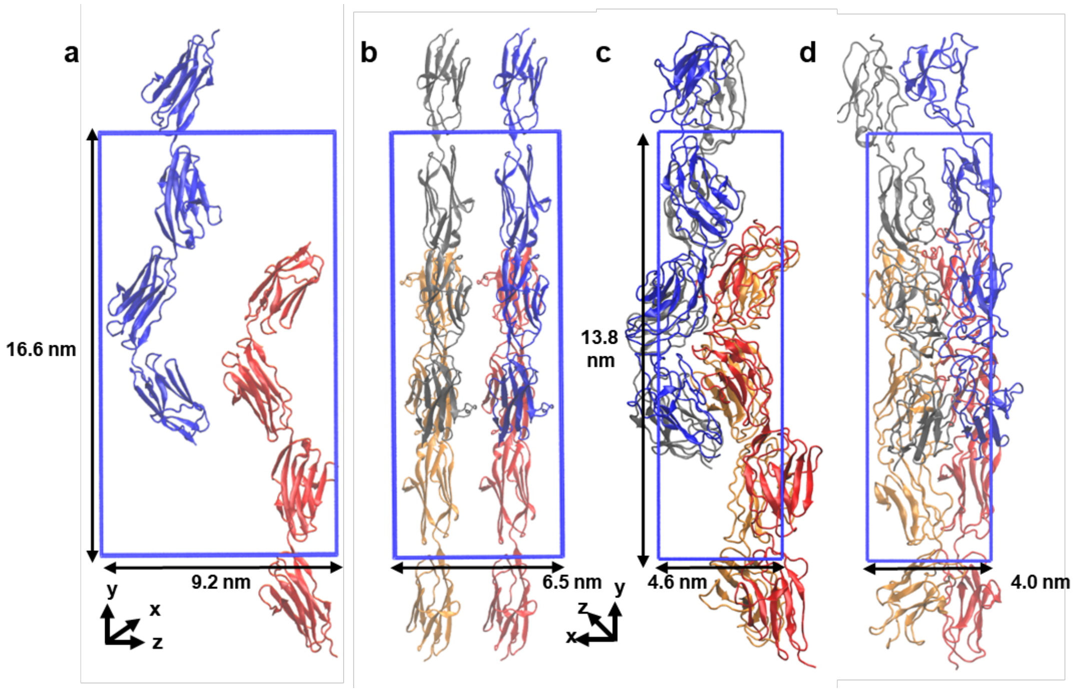

Supplementary Fig. 12. Starting configuration of the molecular dynamics model of the titin fiber and its configuration after equilibration. (a) View from the x-axis of the all-atomistic model of titin fibrils (I67-I70) before equilibration starting from an anti-parallel configuration aligned along the y-axis. The top two Ig domains of the red fibril are closest to the bottom two Ig domains of the blue fibril to create a staggered imbricated arrangement. **(b)** View from the z-axis of the all-atomistic model of titin fibrils (I67-I70) before equilibration. The left two fibrils are shifted replicates of the right two fibrils. Dimensions of the simulation box are 6.5 × 16.6 × 9.2 nm (x, y, z). **(c, d)** Views from the x- and z-axes, respectively of model after equilibration. Dimensions of the simulation box after equilibration are 4.0 × 13.8 × 4.6 nm (x, y, z). We chose this box size to minimize the number of particles simulated (due to limited computational resources) while still yielding a representative system that is informative and reflects the influences of titin interfacial interactions, intrinsic structure, and fibril alignment on the final mechanical properties of the bulk titin fiber. Further discussion of the model setup can be found in Supplementary Note 3.

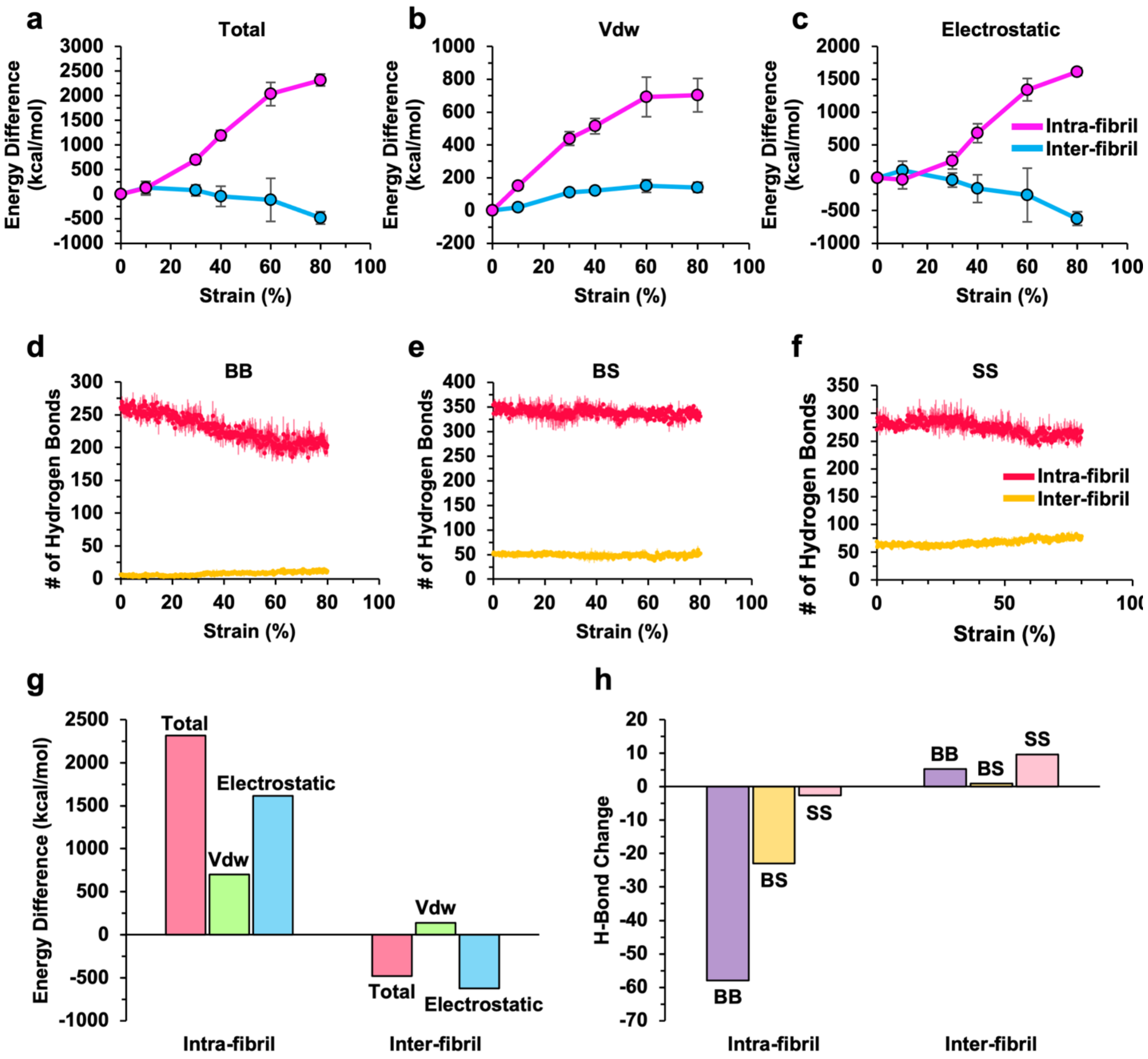

Supplementary Fig. 13. Changes in non-bonded interactions and hydrogen bonds as a function of tensile strain in the titin fiber MD simulation. (a-c) Changes of intra-fibril (pink lines) and inter-fibril (blue lines) total non-bonded (a), Van der Waals (Vdw) (b), and electrostatic (c) energy differences versus tensile strain. Error bars are the standard deviation of three trials. **(d-f)** Changes of intra-fibril (red line) and inter-fibril (yellow line) hydrogen bond numbers, between backbones (BB) (d); between backbones and sidechains (BS) (e); and between sidechains (SS) (f). Error bars (shown as red or yellow bars) are the standard deviation of three trials. **(g)** Total changes

in intra- and inter-fibril non-covalent bonding energies between 0 and 80% strain. **(h)** Total change in number of intra- or inter-fibril hydrogen bonds between 0 and 80% strain. Hydrogen bonds formed between backbones (BB); between backbones and sidechains (BS); and between sidechains (SS). Source data are provided as a Source Data file.

Supplementary Table 1. Measured diameters and mechanical properties of microbially produced UHMW titin fibers. Clamp-to-clamp gauge length for all fibers was 5 mm.

FIBER	Diam. A (µm)	Diam. B (µm)	Diam. C (µm)	Avg. Diameter (µm)	σ (MPa)	E (GPa)	ε (%)	U_T (MJ/m³)
**1**	9.97	9.29	10.43	9.90	360	3.90	53	145
**2**	9.96	9.79	10.25	10.00	345	3.82	52	129
**3**	9.99	10.42	10.42	10.28	345	4.57	50	132
**4**	9.95	9.64	10.91	10.17	344	3.69	53	134
**5**	10.52	9.66	10.00	10.06	368	4.56	50	137
**6**	9.64	10.16	9.65	9.82	367	4.52	48	134
**7**	8.50	9.20	10.16	9.29	438	4.58	49	157
**8**	10.73	9.43	10.06	10.07	372	4.75	52	144
**9**	10.11	10.45	10.42	10.33	358	3.73	52	136
**10**	8.18	9.33	8.99	8.83	426	4.14	35	104
**11**	8.98	8.66	8.82	8.82	436	4.47	41	126
**12**	8.78	9.33	10.55	9.55	375	3.36	39	101
**13**	8.83	8.51	8.82	8.72	442	5.20	36	114
**14**	10.77	10.92	11.08	10.92	313	3.27	54	124

Supplementary Table 2. Parameters extracted from the WAXD image of microbially produced UHMW titin fiber.

Mode of Integration	Peak Index	Peak Center (PC)	FWHM	Intensity (I)
**Equatorial**	(200)	0.584 ($Å^{-1}$)	0.523 ($Å^{-1}$)	107 (a.u.)
	(120)	1.37 ($Å^{-1}$)	0.195 ($Å^{-1}$)	8.73 (a.u.)
	Amorphous 1	1.25 ($Å^{-1}$)	1.22 ($Å^{-1}$)	376 (a.u.)
	Amorphous 2	2.36 ($Å^{-1}$)	1.65 ($Å^{-1}$)	158 (a.u.)
**Meridian**	(200)	0.564 ($Å^{-1}$)	0.565 ($Å^{-1}$)	111 (a.u.)
	(120)	1.37 ($Å^{-1}$)	0.269 ($Å^{-1}$)	5.13 (a.u.)
	(002)	1.97 ($Å^{-1}$)	0.717 ($Å^{-1}$)	29.7 (a.u.)
	Amorphous 1	1.24 ($Å^{-1}$)	1.07 ($Å^{-1}$)	332 (a.u.)
	Amorphous 2	2.47 ($Å^{-1}$)	1.60 ($Å^{-1}$)	147 (a.u.)
**Azimuthal (120)**	(120)	183/370 (°)	29.3/25.3 (°)	497/240 (a.u.)
	(201)	115/235/312/426 (°)	34.3/15.6/22.6/35.1 (°)	336/138/252/528 (a.u.)
	Amorphous	181/363 (°)	91.6/59.4 (°)	1340/1300 (a.u.)
**Azimuthal (200)**	(200)	186/367 (°)	53.6/ 54.2 (°)	164/138 (a.u.)
	Amorphous	187/366 (°)	80.4/70.3 (°)	80.5/50.5 (a.u.)

Supplementary Table 3. Values calculated from WAXD diffraction parameters.

Calculated Parameter	Value	Calculation Method
**a-axis (inter-sheet) D-spacing**	1.08 nm	$\frac{2\pi}{PC_{Equatorial\,(200)}} = \frac{2\pi}{0.584Å^{-1}}$
**b-axis (inter-chain) D-spacing**	0.46 nm	$\frac{2\pi}{PC_{Equatorial\,(120)}} = \frac{2\pi}{1.37Å^{-1}}$
**a-axis (inter-sheet) crystal size**	1.08 nm	$\frac{K \times \lambda}{\arcsin\left(\frac{2 \times \lambda \times FWHM_{Equatorial\,(200)}}{4\pi}\right) \times \cos\left(\arcsin\left(\frac{\lambda \times PC_{Equatorial\,(200)}}{4\pi}\right) \times \frac{360}{2\pi}\right)} = \frac{0.9 \times 0.886Å}{\arcsin\left(\frac{2 \times 0.886Å \times 0.523Å^{-1}}{4\pi}\right) \times \cos\left(\arcsin\left(\frac{0.886Å \times 0.584Å^{-1}}{4\pi}\right) \times \frac{360}{2\pi}\right)}$
**b-axis (inter-chain) crystal size**	2.91 nm	$\frac{K \times \lambda}{\arcsin\left(\frac{2 \times \lambda \times FWHM_{Equatorial\,(120)}}{4\pi}\right) \times \cos\left(\arcsin\left(\frac{\lambda \times PC_{Equatorial\,(120)}}{4\pi}\right) \times \frac{360}{2\pi}\right)} = \frac{0.9 \times 0.886Å}{\arcsin\left(\frac{2 \times 0.886Å \times 0.195Å^{-1}}{4\pi}\right) \times \cos\left(\arcsin\left(\frac{0.886Å \times 1.37Å^{-1}}{4\pi}\right) \times \frac{360}{2\pi}\right)}$
**% Crystallinity**	18%	$\frac{I_{Equatorial(200)} + I_{Equatorial(120)}}{I_{Equatorial(200)} + I_{Equatorial(120)} + I_{EquatorialAmorphous1} + I_{EquatorialAmorphous2}} = \frac{107 + 8.73}{107 + 8.73 + 376 + 158}$
***f*crystal**	0.76	$f_{crystal} = \frac{3\cos^2\varphi - 1}{2}$<br>$\cos^2\varphi = 1 - 0.8 \times \sin^2\left(0.4 \times FWHM_{Equatorial\,(200)}\right) - 1.2 \times \sin^2\left(0.4 \times FWHM_{Equatorial\,(120)}\right) = 1 - 0.8 \times \sin^2\left(0.4 \times \frac{53.6 + 54.2}{2} \times \frac{2\pi}{360}\right) - 1.2 \times \sin^2\left(0.4 \times \frac{29.3 + 25.3}{2} \times \frac{2\pi}{360}\right)$

Supplementary Table 4. Gene sequences used in this study.

Sequence Name	Description	Sequence
4XT	Codon optimized CDS for rabbit soleus titin domains I67-I70	GGTACCCACCACCATCACCATCATCCGCCGTTCTTCGATCTGAAGCCGGTGTCTGTTGAC<br>CTGGCTCTGGGTGAATCTGGCACCTTCAAATGCCACGTGACCGGCACCGCTCCGATCAAG<br>ATTACGTGGGCGAAAGACAACCGCGAGATTCGTCCAGGCGGCAACTACAAAATGACCCT<br>GGTGGAAAACACTGCGACCCTGACCGTCCTGAAAGTGACCAAAGGTGACGCGGGCCAGT<br>ACACTTGTTACGCAAGCAACGTGGCGGGCAAAGATTCTTGCTCTGCACAGCTGGGCGTG<br>CAGGAACCGCCTCGCTTCATTAAAAAGCTGGAGCCGTCCCGCATCGTGAAACAGGACGA<br>ACATACCCGTTATGAATGCAAAATTGGCGGCTCTCCTGAAATCAAAGTACTGTGGTATAA<br>GGACGAAACCGAGATCCAGGAGTCTTCTAAGTTCCGTATGTCTTTCGTGGAGTCTGTTGC<br>CGTCCTGGAAATGTACAACCTGTCCGTGGAGGACTCCGGTGATTACACTTGCGAGGCGC<br>ACAATGCTGCTGGCAGCGCCAGCTCTTCTACCAGCCTGAAAGTTAAAGAACCGCCGGTTT<br>TCCGTAAAAAGCCGCACCCGGTTGAAACTCTGAAAGGTGCCGACGTGCATCTGGAATGT<br>GAACTGCAGGGTACGCCACCGTTTCAGGTTTCCTGGCACAAAGACAAACGTGAACTGCG<br>CTCTGGCAAGAAATACAAAATTATGTCTGAAAACTTCCTGACGTCCATCCACATCCTGAA<br>CGTCGACTCCGCGGATATCGGCGAATATCAATGCAAAGCGTCTAACGATGTTGGCTCTGA<br>CACCTGCGTGGGTTCTATCACCCTGAAAGCCCCGCCTCGTTTCGTGAAGAAACTGTCTGA<br>TATTAGCACGGTCGTTGGCGAAGAGGTTCAGCTGCAGGCTACTATCGAAGGTGCGGAAC<br>CGATCTCCGTTGCATGGTTTAAAGATAAAGGTGAGATCGTACGTGAATCCGATAACATCT<br>GGATTTCCTACTCTGAAAACATCGCGACTCTGCAATTCTCTCGTGCAGAACCGGCGAACG<br>CTGGCAAATACACCTGTCAGATCAAAAACGAAGCGGGTACTCAGGAATGTTTTGCTACC<br>CTGTCTGTTCTGGAATCCGGA
gp41-1 Int^C	N-terminal split intein	ATGGCTAAGACTAAAATGCTGAAAAAAATTCTGAAGATCGAAGAACTGGACGAACGTGA<br>ACTGATTGACATCGAAGTGAGCGGTAACCATCTGTTCTACGCGAACGACATCCTGACCCA<br>CAACTCTTCTAGCGATGTTGGTACC
gp41-1 Int^N	C-terminal split intein	ACCCGTTCTGGTTACTGTCTGGACCTGAAGACCCAGGTGCAGACTCCACAGGGTATGAA<br>AGAAATCTCTAACATCCAGGTTGGTGATCTGGTTCTGTCCAACACTGGCTATAACGAAGT<br>TCTGAACGTTTTCCCGAAATCCAAGAAAAAATCTTACAAAATCACCCTGGAAGATGGCA<br>AAGAAATCATTTGCTCCGAAGAACACCTGTTCCCGACCCAGACGGGCGAAATGAACATT<br>TCCGGCGGTCTGAAGGAAGGTATGTGCCTGTACGTGAAAGAATAA

Supplementary Table 5. Plasmids used in this study.

Plasmid Name	ORI	Promoter	Resistance	Description	Source
pE8k	pColEI	P_{BAD}	Kan^R	BglBricks vector	Lee et al. 2011 [2]
pB6c	pBBR1	P_{LacO1}	Cm^R	BglBricks vector	Lee et al. 2011 [2]
p-1-4XT-1_B	pColEI	P_{BAD}	Kan^R	Plasmid pE8k containing 4XT sequence flanked by complimentary split inteins gp41-1 Int^N and Int^C	This Study
p-1-4XT-1_L	pBBR1	P_{LacO1}	Cm^R	Plasmid pB6c containing 4XT sequence flanked by complimentary split inteins gp41-1 Int^N and Int^C	This Study
p-4XT	pBBR1	P_{LacO1}	Cm^R	Plasmid pB6c containing 4XT sequence without any split inteins	This Study
p-8XT	pBBR1	P_{LacO1}	Cm^R	Plasmid pB6c containing 8XT sequence without any split inteins	This Study
p-12XT	pBBR1	P_{LacO1}	Cm^R	Plasmid pB6c containing 12XT sequence without any split inteins	This Study

Supplementary Table 6. Primer sequences used in this study.

Primer Name	Description	Sequence
prF1	Primer for adding Kpn2I restriction site to 5' end of 4XT sequence. Used in constructing p-8XT and p-12XT.	tttttccggaGCTAGCGGTACCCACCACCATCACC
prF2	Primer for adding NheI restriction site to 5' end of 4XT sequence. Used in constructing p-12XT.	tttttccggaGCTAGCGGTACCCACCACCATCACC
prR1	Primer for maintaining stop codon and BamHI restriction site on 3' end of 4XT sequence. Used in constructing p-8XT and p-12XT.	tttttccggaGGATCCTTATTCCAGAACAGACAGGGTAG
prR2	Primer for adding SpeI restriction site to 3' end of 4XT sequence. Used in constructing p-12XT.	tttttccggaACTAGTTTCCAGAACAGACAGGGTAGC

Supplementary Table 7. Strains used in this study.

Strain Name	Description	Source
NEB10β	F' proA+B+ lacIq Δ(lacZ)M15 zzf::Tn10 (TetR) Δ(ara-leu) 7697 araD139 fhuA ΔlacX74 galK16 galE15 e14- Φ80dlacZΔM15 recA1 relA1 endA1 nupG rpsL (StrR) rph spoT1 Δ(mrr-hsdRMS-mcrBC)	NEB
s-1-4XT-1_B	NEB10β containing plasmid p-1-4XT-1_B	This Study
s-1-4XT-1_L	NEB10β containing plasmid p-1-4XT-1_L	This Study
s-4XT	NEB10β containing plasmid p-4XT	This Study
s-8XT	NEB10β containing plasmid p-8XT	This Study
s-12XT	NEB10β containing plasmid p-12XT	This Study

Supplementary Table 8. Mechanical properties for different microbially produced (blue), natural (green), and manmade (grey) materials. Relevant references are to the right of each value.

Material	Tensile Strength (MPa)	Toughness (MJ/m^3)	Damping capacity (%)
Microbially Produced Titin (this work)	378	130	81
Bacterial cellulose (BC)	0.12-0.68 [3]	0.016-0.080 [3]	--
GB1-resilin muscle mimic	0.026-0.057 [4]	0.004-0.014 [4]	13-18 [4]
Poly(3-hydroxybutyrate-co-3-hydroxyvalerate) (PHBV)	38.8-44.6 [5]	*1.2* [5]*	--
Poly(3-hydroxybutyrate-co-3-hydroxyvalerate)–Poly(ε-caprolactone) (PHBV–PCL)	36.2-39.2 [5]	*1.7-8.9* [5]*	--
Polyhydroxybutyrate (PHB)	14.3-32.1 [6]	0.244-2.00 [6]	--
Poly(3-hydroxybutyrate-co-4-hydroxyvalerate) (P34HB)	17-65 [7]	--	40.6 [8]
Polyhydroxybutyrate–polypropylene (PHB–PP)	24.5-27.5 [9]	*0.21-0.99* [9]*	--
Poly(lactic acid) (PLA)	21-60 [10]	2 [11]	--
ε-Poly-L-lysine–Lignosulfonate (ε-PL–Lignosulfonate)	3.1-27.9 [12]	0.4-8.4 [12]	--
Bone (mineralized collagen)	110-875 [13]	4 [14]	--
Cotton	205-778 [13]	4.27-30.1 [15]	45-72 [16]
Elastin	0.306-1.38 [13]	2 [14]	24 [16]
Resilin	3.03-4.25 [13]	4 [14]	3 [16]
Silkworm silk	540-592 [17]	47-64 [14]	48-65 [16]
Spider silk (dragline)	544.59-1469.34 [18]	47.85-230.02 [18]	41-75 [16]
Tendon (collagen)	45.9-182 [13]	1-1.78 [19]	3-20 [19]
Viscose/rayon	304-662 [15]	32.0-40.1 [15]	65-80 [16]
Wool	37.4-206 [13]	24.3-47.6 [15]	31-65 [16]
Carbon fiber (CF)	4000 [14]	25 [14]	--
Kevlar	3600 [17]	60 [14]	--
Nylon	750-950 [17]	63.4-107 [15]	5-40 [16]
Polypropylene (PP)	20.9-37.1 [20]	170 [21]	8-35 [16]
Polyurethane (PU)	18.8 [22]	79.5 [22]	--
Steel	1650 [17]	6 [14]	--
Synthetic rubber	50 [23]	100 [23]	76.2 [24]

*For materials that did not report toughness data, a toughness "upper limit" was calculated using the reported mechanical properties (see Supplementary Note 2).

Supplementary Note 1. CD spectra were analyzed with the BeStSel Single Spectrum Analysis and Fold Recognition tool (http://bestsel.elte.hu/index.php, last accessed Aug 9, 2020). In brief, this algorithm estimates the secondary structure content of the sample spectrum by fitting to spectra of proteins with known structures. All proteins in the database are indexed based on proportions of eight different secondary structure elements (regular alpha-helix, distorted alpha-helix, left-twisted antiparallel beta-strand, relaxed antiparallel beta-strand, parallel beta strand, turn, and others). Each structure is represented as a point in an eight-dimensional secondary structure space. The algorithm reports the predicted proportion of eight secondary structural elements for the sample spectrum and gives a list of the top ten structures closest to the predicted sample structure in this eight-dimensional space. Details can be found in the associated publication[25].

Supplementary Note 2. For materials that did not have a reported toughness, a toughness "upper limit" was calculated using the reported values for tensile strength, Young's modulus, and elongation at break. Because ultimate tensile strength defines the maximum measured stress, elongation at break defines the maximum measured strain, and Young's modulus defines the steepest measured slope of the curve (during the elastic regime), the following equations derived from the figure below can be used to calculate the maximum possible toughness value for any set of those three mechanical properties for a given material:

$$\theta_1 = \tan^{-1}(E/1)$$

$$\theta_2 = \tan^{-1}(1/E)$$

$$\sigma_1 = \varepsilon_{max} * \tan\theta_1$$

$$\sigma_2 = \sigma_1 - \sigma_{max}$$

$$\varepsilon_1 = \sigma_2 * \tan\theta_2$$

$$\text{Toughness upper limit} = (\varepsilon_{max} * \sigma_1)/2 - (\varepsilon_1 * \sigma_2)/2$$

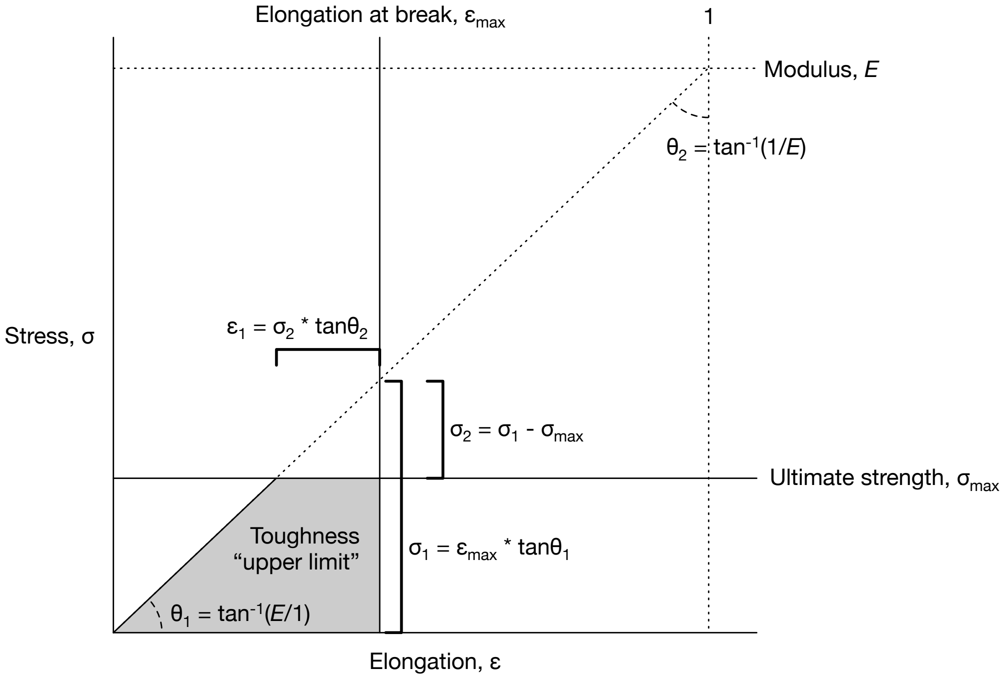

Supplementary Note 3. To construct a representative model of the macro-scale titin fiber, we first assumed that the titin domains do not unfold appreciably during the fiber spinning process, as proteins are known to remain folded even under high shear flow[26,27]. We then employed the protein docking GRAMM-X web server[28] in attempt to establish an initial assembly of two titin chains (figure (a) below). However, the energy minimized structures predicted using protein docking did not yield conformations with aligned chains as seen in the experimental data of our fibers. This is likely because the docking results obtain a local minimum on the basis of equilibrium conditions and cannot take into account the surrounding protein chains and mechanical microenvironment present during spinning, which can induce greater packing and orientation. Specifically, high shear flow can significantly impact the supramolecular assembly of proteins. For example, amyloid proteins can transfer from spherical aggregates in low shear flow ($\dot{\gamma}\sim 40/s$) to thick fibers in high shear flow ($\dot{\gamma}\sim 400/s$)[29]. The shear flow in our experiment is calculated as $\dot{\gamma}\sim 500/s$, therefore we assume that the titin chains will be well-aligned along the fiber axis. This assumption is supported by our experimental results from Raman and X-ray diffraction analysis. This, along with the observed large initial modulus of our real titin fibers, leads us to believe that the bending motion of individual titin chains is likely hindered due to lateral packing into bundles through close interactions between chain surfaces. Hence, we applied a lateral pressure to compress the initially loosely assembled titin fiber in our simulation. Additionally, we also noticed the importance of the alignment of single titin chains in the simulated titin fiber. The staggered assembly pattern allows the shear force transmission between adjacent chains, which ensures the high stiffness and toughness of the bulk fiber. In comparison, if the chains stack face-to-face or in any other orientation, then the stress cannot transmit across the fiber, resulting in a very weak fiber (figure (c) below). Therefore, we chose a representative model with two Ig domains stacked into a well-

packed configuration. The inter-fibril interaction energy calculated from this configuration (-1415 kcal/mol, figure (b) below) is more favorable than the structures obtained from GRAMM-X (-1009 kcal/mol, figure (a) below).

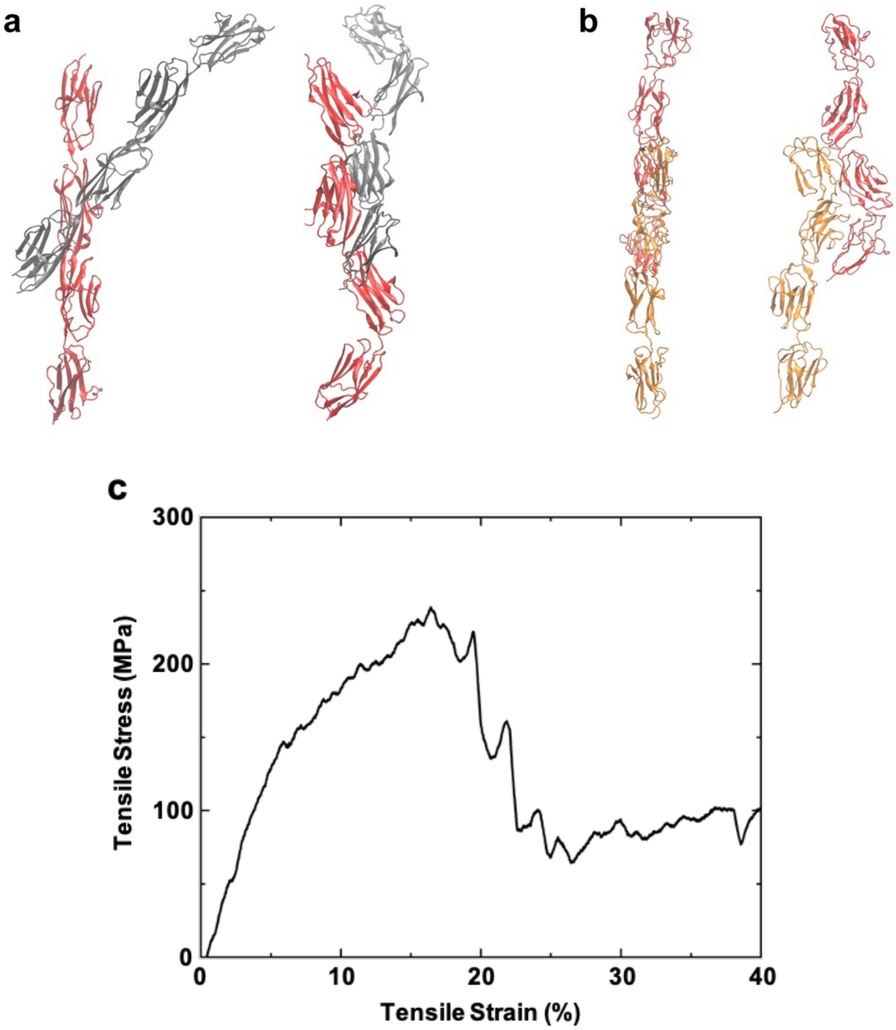

(a) Equilibrated conformation calculated from GRAMM-X web server. (b) Aligned conformation from our model. (c) Tensile stress-strain curve of face-to-face stacked titin fiber.

Between interfaces of equilibrated titin chains, there exist Vdw and electrostatic interactions, which change slightly during the fiber stretching. We also calculated the solvent-accessible surface (SASA) of a titin chain buried in surrounding titin chains using VMD[30]. SASA of the single chain is S1, SASA of the single chain with surrounding chains is S2, and SASA of the complex without

the single chain is S3. The single chain interaction area ratio is defined by (S3+S1-S2)/2S1[31], which is calculated as 74% for our titin. In contrast, if the chains are not compressed during equilibration, the interaction area ratio is calculated as 52%. Hence, the titin chains in our model interact extensively with other chains.

Supplementary Note 4. To compare our approach with constant velocity Steered MD (SMD), we first used LAMMPS with the 'fix deform' command to stretch a single titin chain (with the top and bottom of the chain linked across the periodic box boundary, such that this represents an infinite chain with repeats in image cells). The tensile strain rate was 1×10^8/s, which can be related to a pulling rate of 1.2 m/s that is comparable to previous studies with MD. Figure (a) below is the representative single titin fibril tensile force-displacement curve we calculated using this approach. The overall shape of the curve matches the saw-tooth pattern from experiments and other SMD simulations for single chains[32,33], with gradual increases of force and sudden drops after peak force that indicate the cooperative unfolding of titin domains. The distance to fully unfold I67, the first Ig domain to unfold, is about 25 nm, which matches well with previous SMD simulations[33]. The sequential unfolding of domains, shown in (c) below, is also similar to previous SMD simulations. Furthermore, to validate our approach, we also used SMD in NAMD to pull a single titin chain with a pulling velocity of 1.2 m/s; the measured curve of tensile force (figure (b) below) matches well the result measured with LAMMPS.

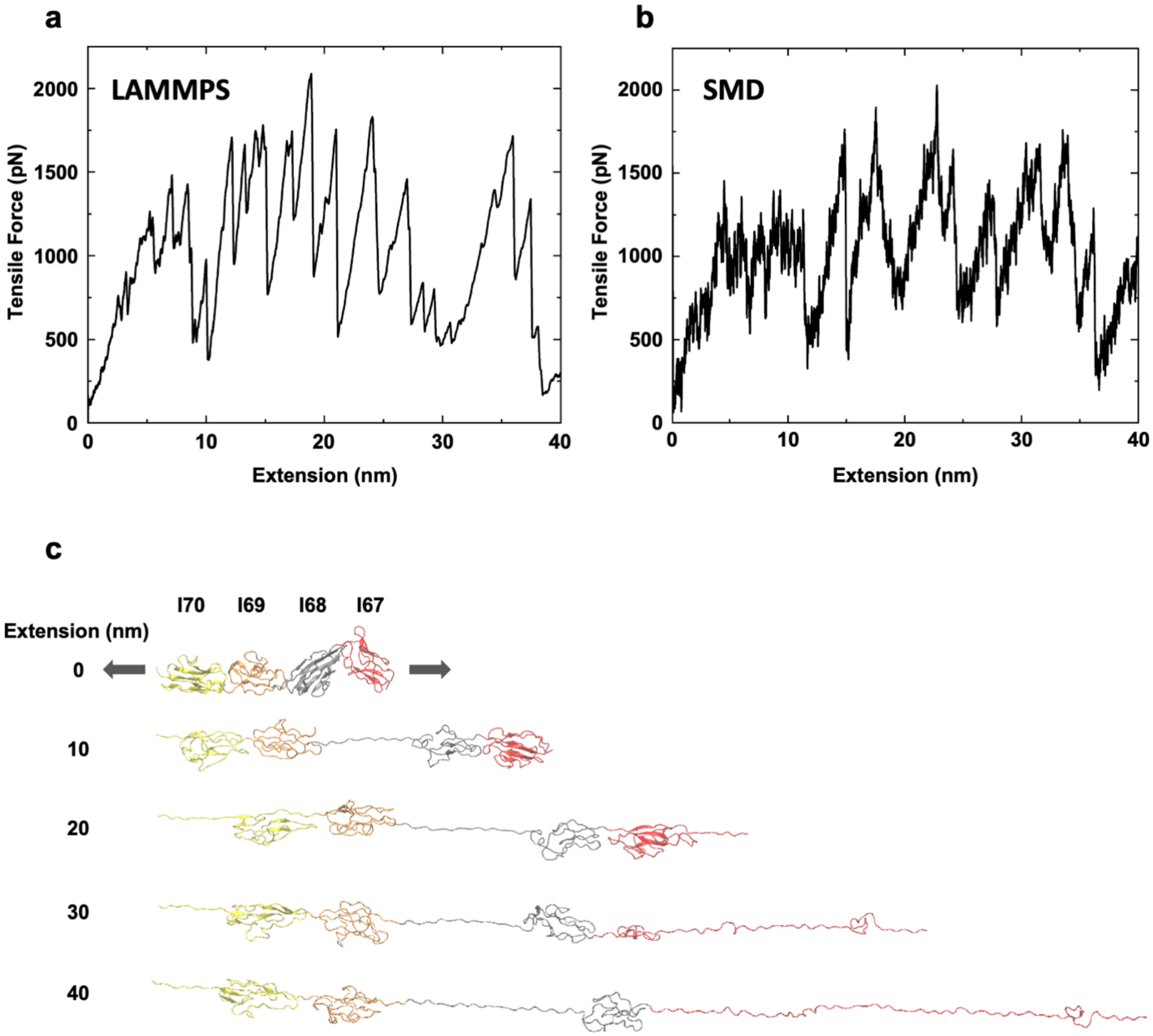

(a) Tensile force-displacement curve of a single titin fibril simulated by LAMMPS. (b) Tensile force-displacement curve of a single titin fibril simulated by NAMD SMD. (c) Schematic of the stretching of a single titin fiber.

Supplementary References:

1. Von Castelmur, E. *et al.* A regular pattern of Ig super-motifs defines segmental flexibility as the elastic mechanism of the titin chain. *Proc. Natl. Acad. Sci. U. S. A.* **105**, 1186–1191 (2008).

2. Lee, T. S. *et al.* BglBrick vectors and datasheets: A synthetic biology platform for gene expression. *J. Biol. Eng.* **5**, 15–17 (2011).

3. Chen, S.-Q., Lopez-Sanchez, P., Wang, D., Mikkelsen, D. & Gidley, M. J. Mechanical properties of bacterial cellulose synthesised by diverse strains of the genus Komagataeibacter. *Food Hydrocoll.* **81**, 87–95 (2018).

4. Lv, S. *et al.* Designed biomaterials to mimic the mechanical properties of muscles. *Nature* **465**, 69–73 (2010).

5. Zytner, P., Wu, F., Misra, M. & Mohanty, A. K. Toughening of Biodegradable Poly(3-hydroxybutyrate-co-3-hydroxyvalerate)/Poly(ε-caprolactone) Blends by In Situ Reactive Compatibilization. *ACS Omega* **5**, 14900–14910 (2020).

6. Anbukarasu, P., Sauvageau, D. & Elias, A. Tuning the properties of polyhydroxybutyrate films using acetic acid via solvent casting. *Sci. Rep.* **5**, 17884 (2015).

7. Saito, Y., Nakamura, S., Hiramitsu, M. & Doi, Y. Microbial synthesis and properties of poly(3-hydroxybutyrate-co-4-hydroxybutyrate). *Polym. Int.* **39**, 169–174 (1996).

8. Zhu, C. & Chen, Q. Polyhydroxyalkanoate-Based Biomaterials for Applications in Biomedical Engineering. *Advanced Healthcare Materials* 439–464 (2014). doi:doi:10.1002/9781118774205.ch12

9. Pachekoski, W. M., Agnelli, J. A. M. & Belem, L. P. Thermal, mechanical and morphological properties of poly (hydroxybutyrate) and polypropylene blends after

processing . *Materials Research* **12**, 159–164 (2009).

10. Farah, S., Anderson, D. G. & Langer, R. Physical and mechanical properties of PLA, and their functions in widespread applications — A comprehensive review. *Adv. Drug Deliv. Rev.* **107**, 367–392 (2016).
11. Jing, F. & Hillmyer, M. A. A Bifunctional Monomer Derived from Lactide for Toughening Polylactide. *J. Am. Chem. Soc.* **130**, 13826–13827 (2008).
12. Ushimaru, K., Hamano, Y., Morita, T. & Fukuoka, T. Moldable Material from ε-Poly-l-lysine and Lignosulfonate: Mechanical and Self-Healing Properties of a Bio-Based Polyelectrolyte Complex. *ACS Omega* **4**, 9756–9762 (2019).
13. Wegst, U. G. K. & Ashby, M. F. The mechanical efficiency of natural materials. *Philos. Mag.* **84**, 2167–2186 (2004).
14. Agnarsson, I., Kuntner, M. & Blackledge, T. A. Bioprospecting Finds the Toughest Biological Material: Extraordinary Silk from a Giant Riverine Orb Spider. *PLoS One* **5**, e11234 (2010).
15. Matsumoto, K. Kim, W. Lee, K. Toughness of Textile Fibers. *J. Text. Mach. Soc. Japan* **47**, 66–71 (1994).
16. Wu, Y. *et al.* Bioinspired supramolecular fibers drawn from a multiphase self-assembled hydrogel. *Proc. Natl. Acad. Sci. U. S. A.* **114**, 8163–8168 (2017).
17. Yarger, J. L., Cherry, B. R. & van der Vaart, A. Uncovering the structure–function relationship in spider silk. *Nat. Rev. Mater.* **3**, 18008 (2018).
18. Swanson, B. O., Blackledge, T. A., Summers, A. P. & Hayashi, C. Y. Spider Dragline Silk: Correlated and Mosaic Evolution in High-Performance Biological Materials. *Evolution (N. Y).* **60**, 2539–2551 (2006).

19. Pollock, C. M. & Shadwick, R. E. Relationship between body mass and biomechanical properties of limb tendons in adult mammals. *Am. J. Physiol. - Regul. Integr. Comp. Physiol.* **266**, (1994).

20. Ashby, M. F. Chapter 4 - Material Property Charts. in (ed. Ashby, M. F. B. T.-M. S. in M. D. (Fourth E.) 57–96 (Butterworth-Heinemann, 2011). doi:https://doi.org/10.1016/B978-1-85617-663-7.00004-7

21. Keskin, R. & Adanur, S. Improving Toughness of Polypropylene with Thermoplastic Elastomers in Injection Molding. *Polym. Plast. Technol. Eng.* **50**, 20–28 (2011).

22. Venkatesan, H., Hu, J. & Chen, J. Bioinspired Fabrication of Polyurethane/Regenerated Silk Fibroin Composite Fibres with Tubuliform Silk-Like Flat Stress–Strain Behaviour. *Polymers (Basel).* **10**, 333 (2018).

23. Omenetto, F. G. & Kaplan, D. L. New Opportunities for an Ancient Material. *Science (80-.).* **329**, 528 LP – 531 (2010).

24. Feng, X. *et al.* A novel method for constitutive characterization of the mechanical properties of uncured rubber. *J. Elastomers Plast.* **48**, 523–534 (2015).

25. Micsonai, A. *et al.* Accurate secondary structure prediction and fold recognition for circular dichroism spectroscopy. *Proc. Natl. Acad. Sci. U. S. A.* **112**, E3095–E3103 (2015).

26. Jaspe, J. & Hagen, S. J. Do Protein Molecules Unfold in a Simple Shear Flow? *Biophys. J.* **91**, 3415–3424 (2006).

27. Phillips, J. C. Scalable molecular dynamics with NAMD. *J. Comput. Chem.* **26**, (2005).

28. Tovchigrechko, A. & Vakser, I. A. GRAMM-X public web server for protein–protein docking. *Nucleic Acids Res.* **34**, W310–W314 (2006).

29. Foderà, V., Pagliara, S., Otto, O., Keyser, U. F. & Donald, A. M. Microfluidics Reveals a Flow-Induced Large-Scale Polymorphism of Protein Aggregates. *J. Phys. Chem. Lett.* **3**, 2803–2807 (2012).

30. Knapp, B., Lederer, N., Omasits, U. & Schreiner, W. vmdICE: A plug-in for rapid evaluation of molecular dynamics simulations using VMD. *J. Comput. Chem.* **31**, 2868–2873 (2010).

31. Mishra, S. Computational prediction of protein-protein complexes. *BMC Res. Notes* **5**, 495 (2012).

32. Rico, F., Gonzalez, L., Casuso, I., Puig-Vidal, M. & Scheuring, S. High-Speed Force Spectroscopy Unfolds Titin at the Velocity of Molecular Dynamics Simulations. *Science (80-.).* **342**, 741 LP – 743 (2013).

33. Hsin, J., Strümpfer, J., Lee, E. H. & Schulten, K. Molecular Origin of the Hierarchical Elasticity of Titin: Simulation, Experiment, and Theory. *Annu. Rev. Biophys.* **40**, 187–203 (2011).
